# Supplementary material for: Bioinspired graphene membrane with temperature tunable channels for water gating and molecular separation
Source: Nat Commun. 2017 Dec 8;8:2011. doi: 10.1038/s41467-017-02198-5 (PMC5722819; doi:10.1038/s41467-017-02198-5)
Supplement: Supplementary file 1 — Supplementary Information [file 41467_2017_2198_MOESM1_ESM.pdf]

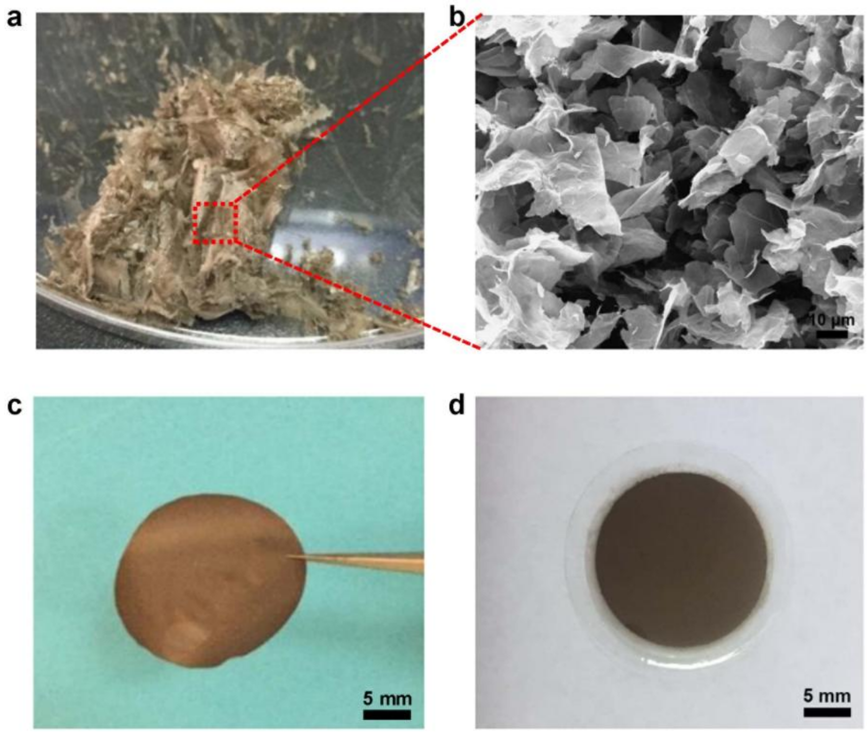

Supplementary Figure 1. Characterization of P-GOM and GOM. a) Digital photo and b) SEM image of P-GO obtained after freezing dry. c) Digital photo of a P-GOM peeled off from substrate. d) Digital photo of a GOM on AAO substrate.

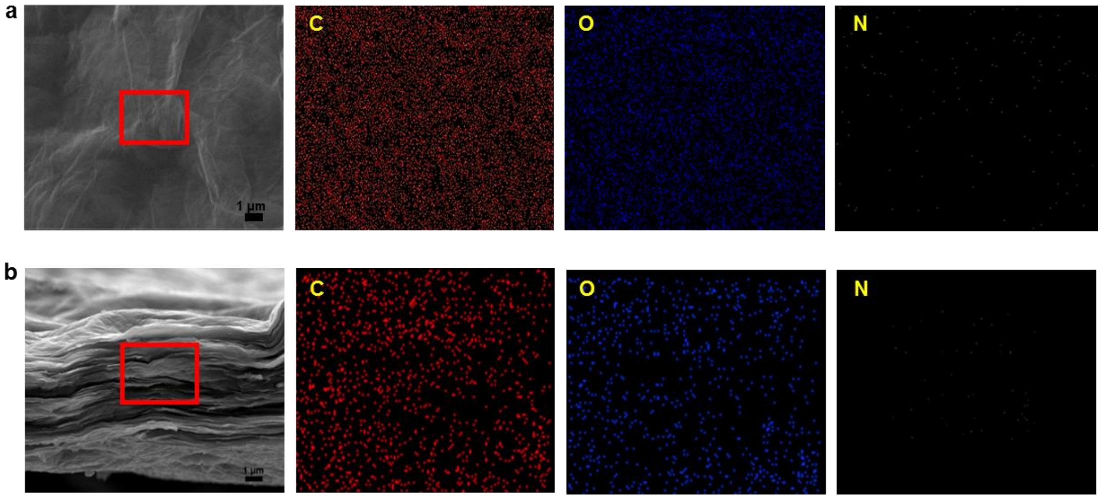

Supplementary Figure 2. SEM images of the GOM. a) the wrinkled surface and b) stacking cross-section of a GOM, the corresponding EDS maps indicated that nitrogen is barely existed on GOM.

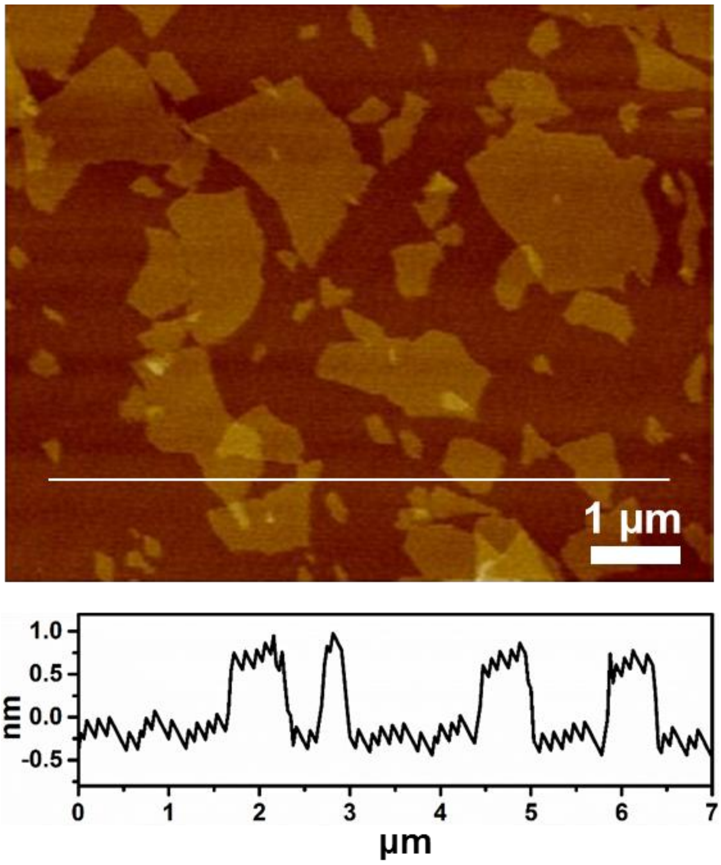

Supplementary Figure 3. AFM image of GO, the thickness of GO sheet is about 0.8 nm.

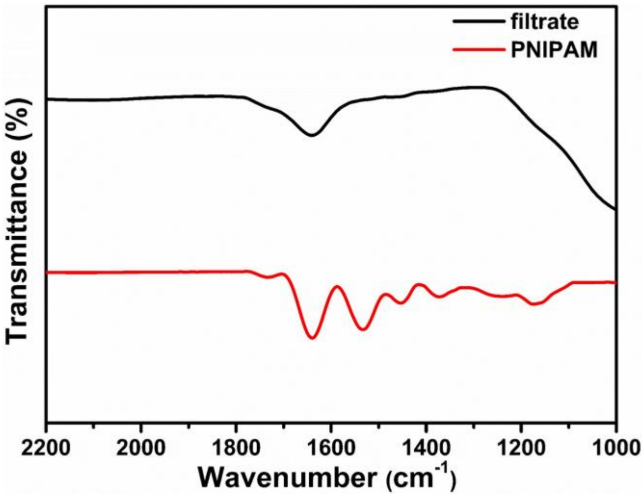

Supplementary Figure 4. ATR-FTIR spectra of the pure PNIPAM and the filtrate obtained when the P-GOMs formed through the pressure-driven filtration, indicating no PNIPAM detached from the P-GOM when it formed. The peak at 1640 cm^{-1} in the spectrum of filtrate is attributed to the bending mode of water[1].

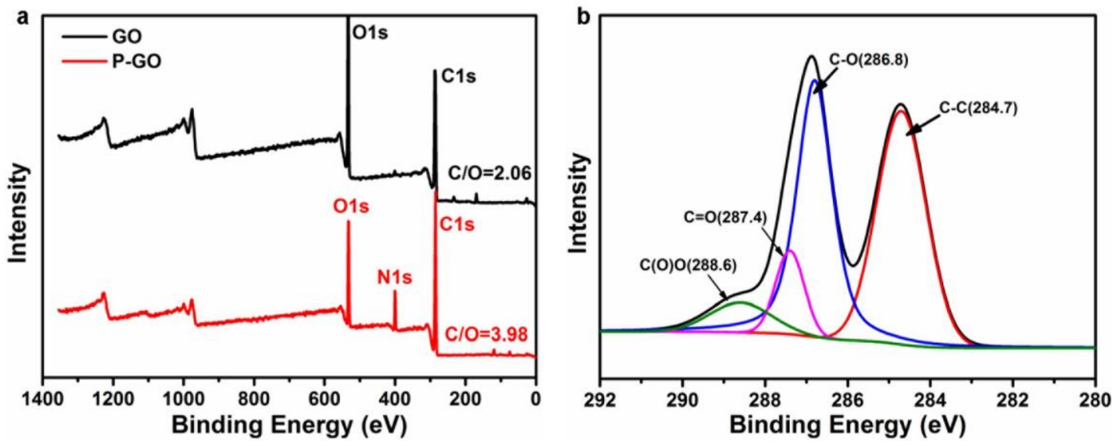

Supplementary Figure 5. XPS Characterization of GO and P-GO. a) The survey scan XPS spectra of GO and P-GO show the peak of N which is characteristic element of PNIPAM. The C/O atomic ratio increased from 2.06 (GO) to 3.98 (P-GO) also supports the successful grafting of PNIPAM chains onto GO sheets. b) XPS spectrum of GO, of which C 1s region fitting into four peaks at 284.7, 286.8, 287.4 and 288.6 eV, representing C-C, C-O, C=O and C(O)O, respectively.

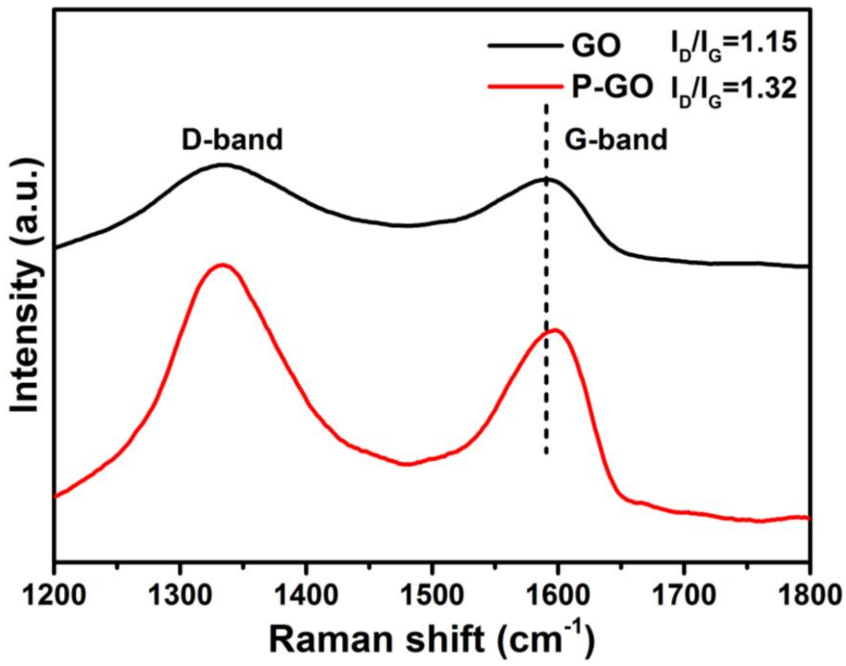

Supplementary Figure 6. Raman characterizations of GO and P-GO. The G band at 1580 cm^{-1} was assigned to the vibration of sp^2 bonded carbon atoms, and the D band at 1350 cm^{-1} was assigned to the vibration of carbon atoms with dangling bonds in plane terminations of disordered graphite, indicating the formation of sp^3 carbon atoms. The I_D/I_G intensity ratio increased from 1.15 (GO) to 1.32 (P-GO), demonstrating the increase of sp^3 carbon structure after PNIPAM grafting. The blue shift of G band from 1580 cm^{-1} to about 1600 cm^{-1} may due to the deteriorated alternate pattern of single-double bonds within the sp^2 carbon sheets caused by the tethering of PNIPAM chain on GO sheet via free radical polymerization[2].

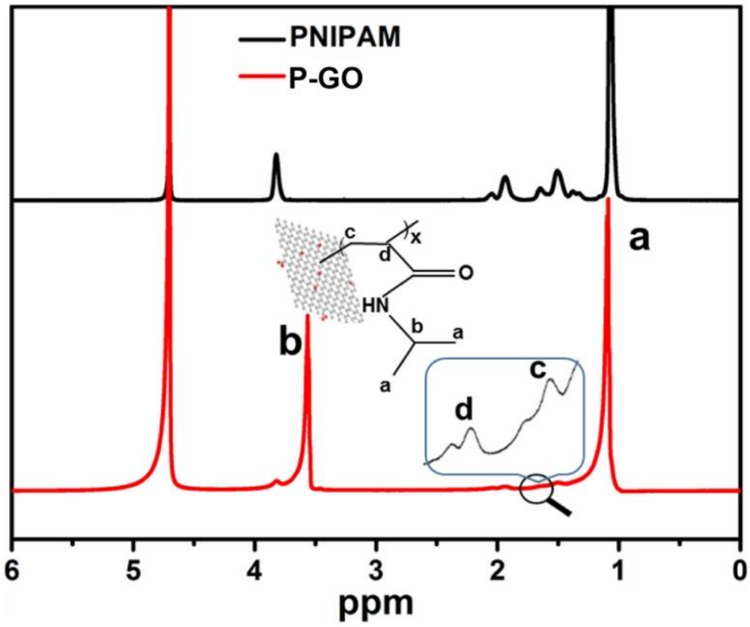

Supplementary Figure 7. ^{1}H NMR spectra of PNIPAM and P-GO in D_2O. For PNIPAM polymer, the ^{1}H NMR spectra clearly shows the characteristic signals of PNIPAN side chains around $\delta = 1.0$, 1.5, 1.9 and 3.8 ppm. After the PNIPAM was grafted to GO sheets, diamagnetic ring currents in GO lead to the upfield shifts of the proton peaks in CH (b) and CH (d) groups, changing from $\delta = 3.8$ to $\delta = 3.6$ and $\delta = 1.9$ to $\delta = 1.8$. The appearance and upfield shifts of these PNIPAM characteristic signals demonstrates that the PNIPAM chains had been successfully grafted to the GO sheets.

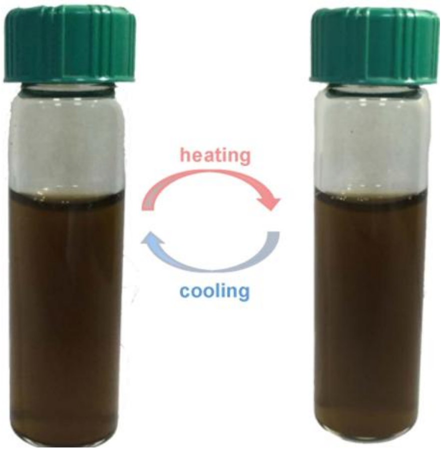

Supplementary Figure 8. The GO aqueous dispersion remains unchanged before and after heating, indicating GO does not have the temperature-responsive property.

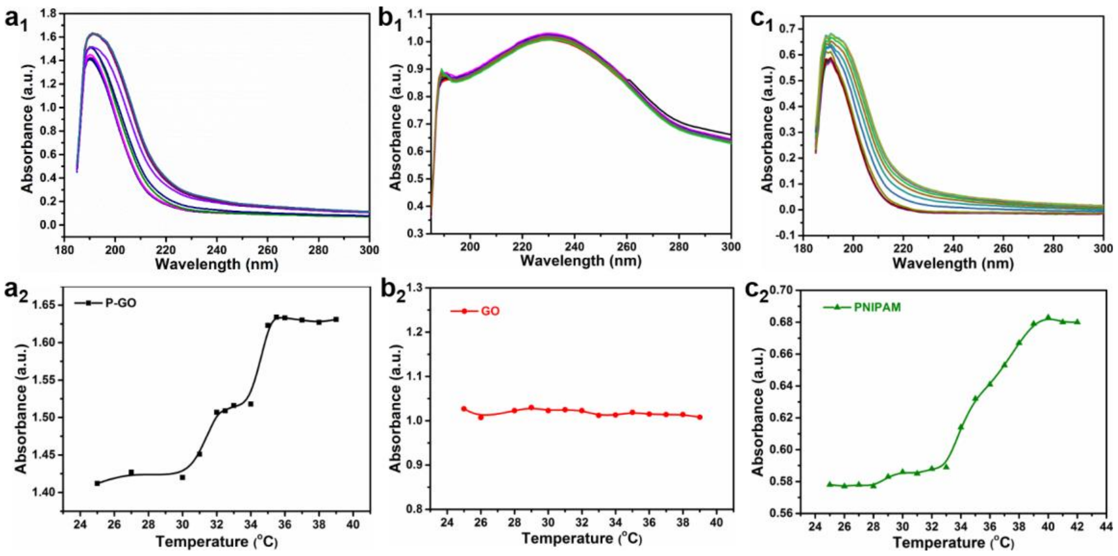

Supplementary Figure 9. The variable-temperature UV-vis spectra of a_1, a_2) P-GO, b_1, b_2) GO and c_1, c_2) PNIPAM, showing the LCST of P-GO is about 30℃, lower than that of PNIPAM.

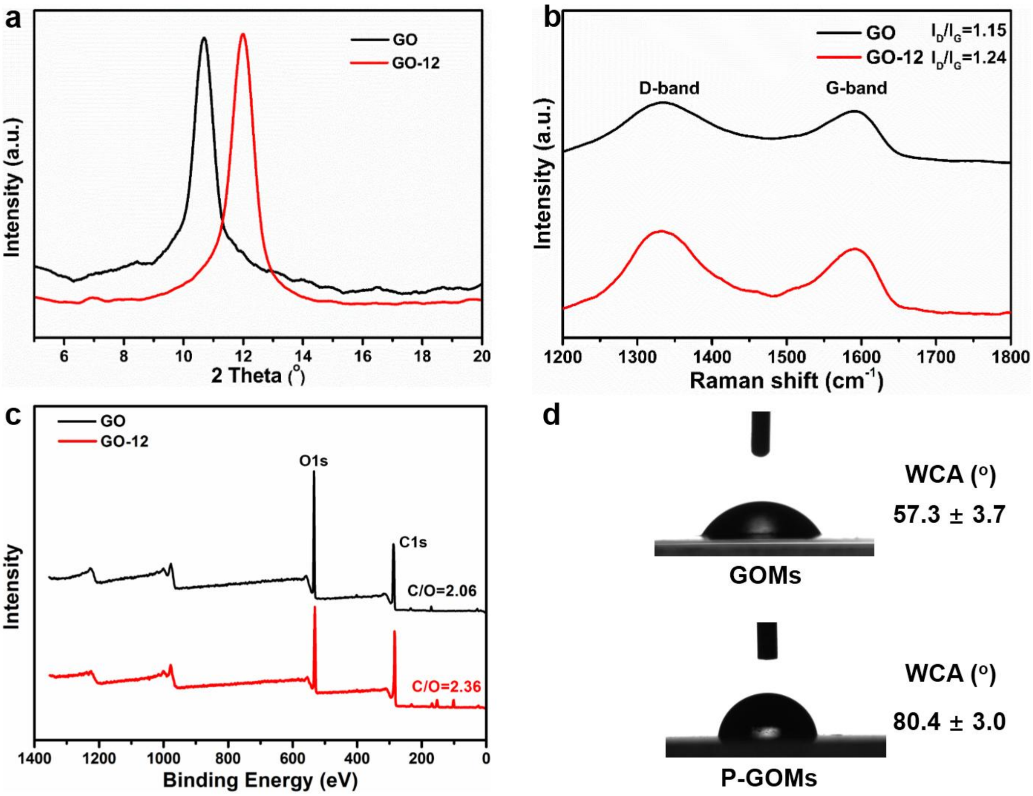

Supplementary Figure 10. Characterizations of GO and GO-12. a) XRD patterns of GO and GO-12 (GO dispersed in 50 mL DMF without NIPAM and AIBN was subjected to 65℃ for 12 h same as the polymerization conditions. The product obtained was named GO-12). After reaction for 12 h, the diffraction peak of GO shifted to higher angles, indicating the decrease of d-spacing, which was due to the partially reduction of GO. b) Raman characterizations of GO and GO-12. The I_D/I_G intensity ratio increased from 1.15 (GO) to 1.24 (GO-12), demonstrating the decrease in the average size of the sp^2 domains upon reduction[3]. c) The survey scan XPS spectra of GO and GO-12 show the C/O atomic ratio increased from 2.06 (GO) to 2.36 (GO-12), demonstrating the reduction of GO. d) The WCAs on the GOMs were 57.3 ± 3.7°, smaller than that on P-GOMs which were 80.4 ± 3.0° although PNIPAM was hydrophilic at room temperature (25℃), indicating that GO in P-GOMs became hydrophobic, further confirming the partially reduction of GO during the polymerization reaction.

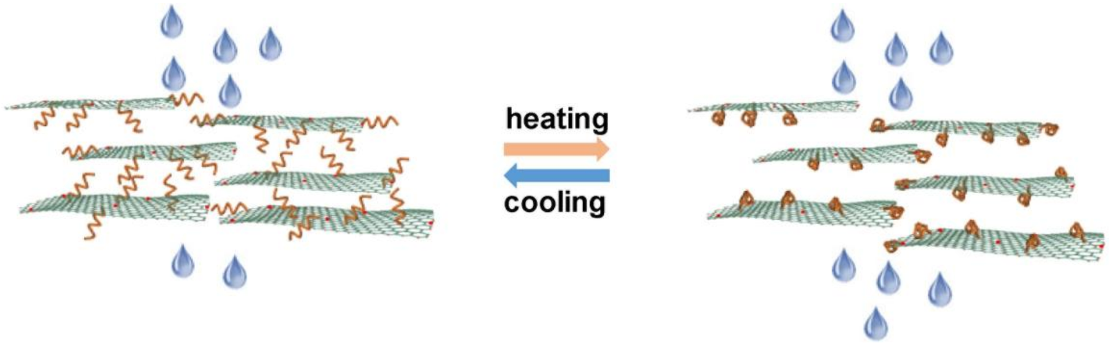

Supplementary Figure 11. Schematic diagram of the positive temperature-responsive property of GO blending with PNIPAM membranes. Researchers had physically blended chemically reduced GO with PNIPAM to construct the thermal responsive graphene membrane, in which the chemically reduced GO and PNIPAM were connected by supramolecular interaction[4]. It showed a positive temperature-response gating behavior. For the P-GOM, it was composed of PNIPAM covalently bound GO *via* free radical polymerization. The covalently bound interactions between PNIPAM chains and GO endowed the membrane negative temperature-response gating behavior.

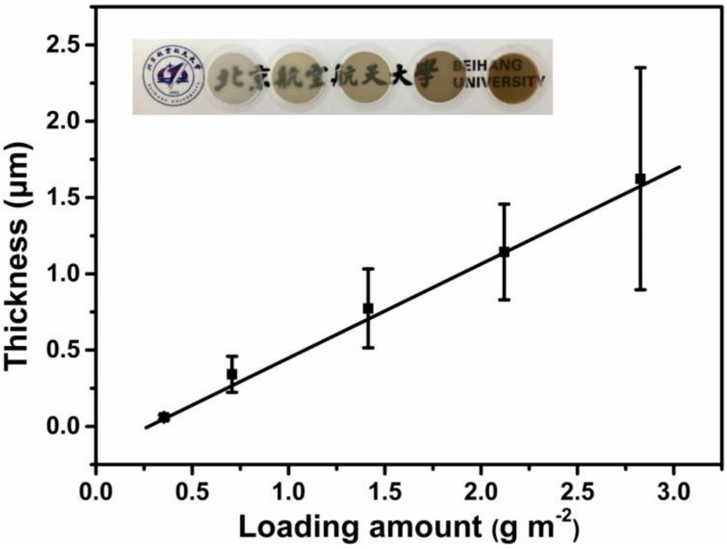

Supplementary Figure 12. The thickness variations of P-GOMs with different loading amounts of P-GO aqueous dispersion. Inset is digital photos of P-GOMs with thickness increasing from left to right.

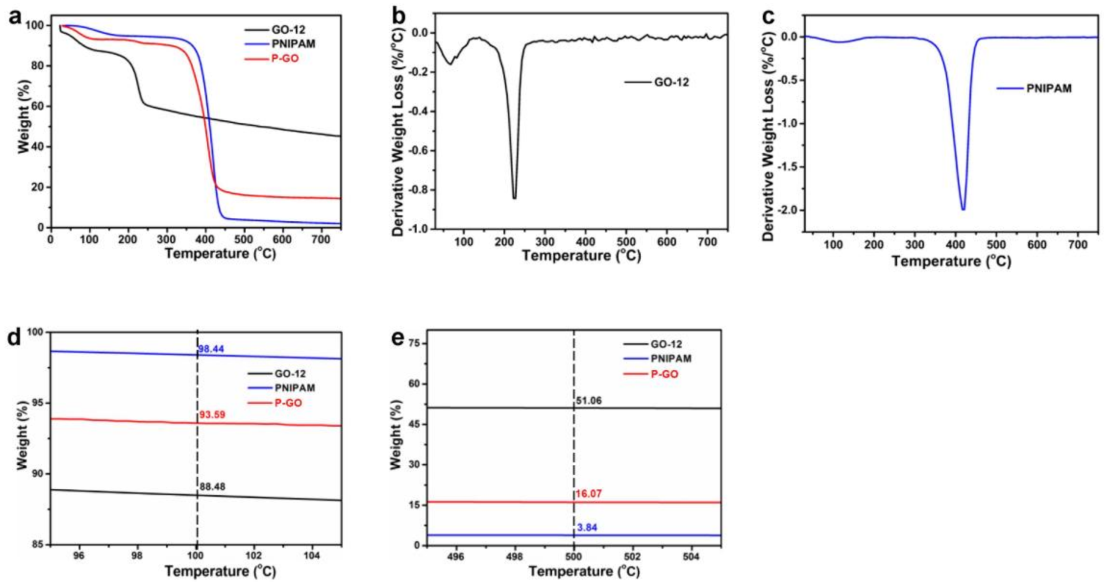

Supplementary Figure 13. a) Thermogravimetric analysis (TGA) curves of GO-12, PNIPAM, and P-GO. The differential thermogravimetry (DTG) curves of b) GO-12 and c) PNIPAM. The high resolution TGA curves at d) ~ 100℃ and e) ~ 500℃.

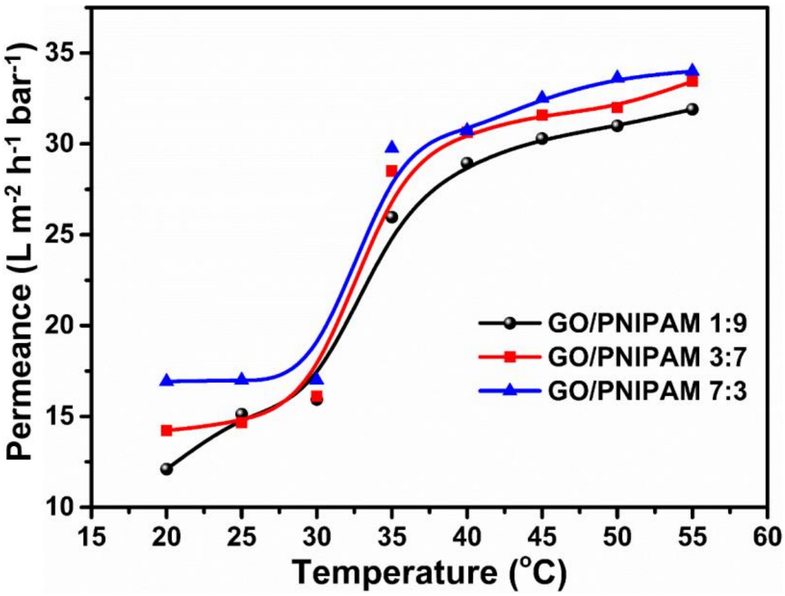

Supplementary Figure 14. Temperature dependent water permeance curves of GO blending with PNIPAM membranes composed with different GO/PNIPAM ratios (1:9, 3:7, and 7:3), all showing the positive temperature-responsive coefficients.

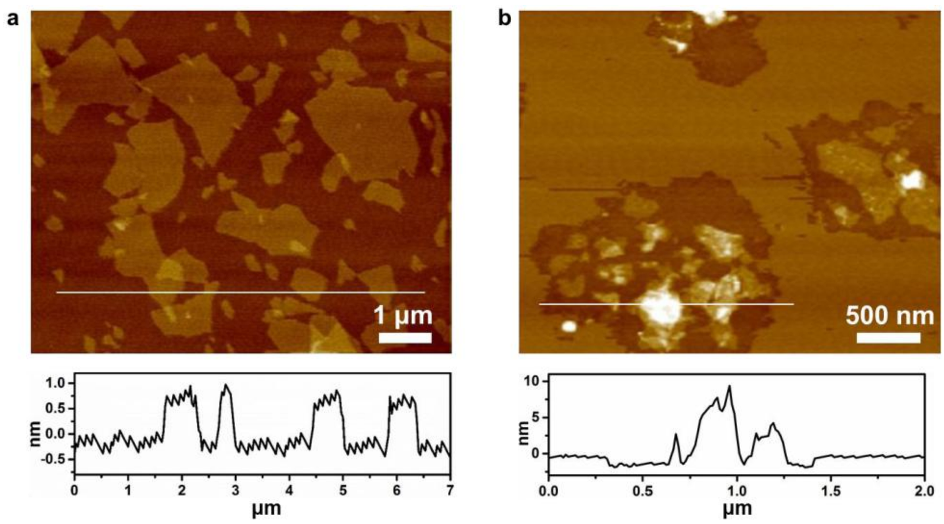

Supplementary Figure 15. AFM images of a) GO and b) P-GO at 25℃. Both of the samples were obtained by spin-coating (2000 rpm) the diluted GO or P-GO aqueous dispersion (~ 0.02 mg/mL) on freshly cleaved mica. The single and stacked P-GO sheets demonstrate that when synthetized the P-GO during the FRP, the PNIPAM chains (1) grew from one GO sheet and terminated freely at the bulk solution or (2) terminated at another GO sheet. There are no stacked GO sheets as the control experiment.

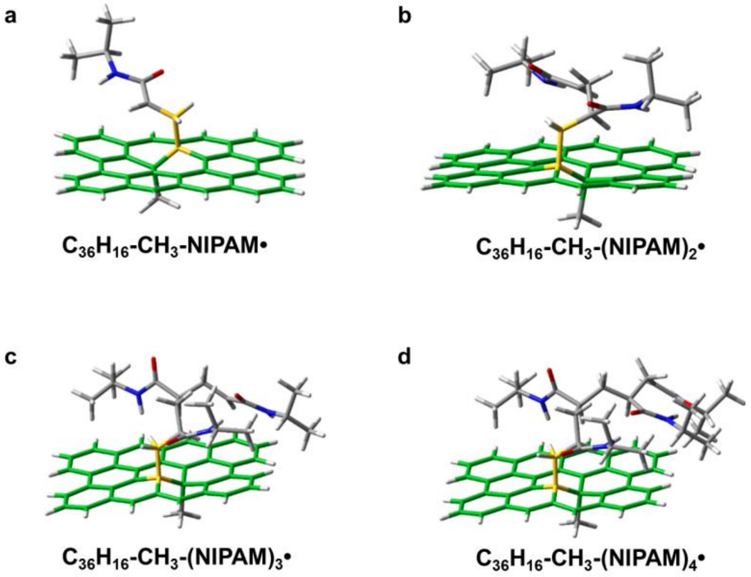

Supplementary Figure 16. a-d) Optimized geometries of the products in reactions (1a) $C_{36}H_{16}$-CH_3-NIPAM•, (2a) $C_{36}H_{16}$-CH_3-$(NIPAM)_2$•, (3a) $C_{36}H_{16}$-CH_3-$(NIPAM)_3$• and (4a) $C_{36}H_{16}$-CH_3-$(NIPAM)_4$•.

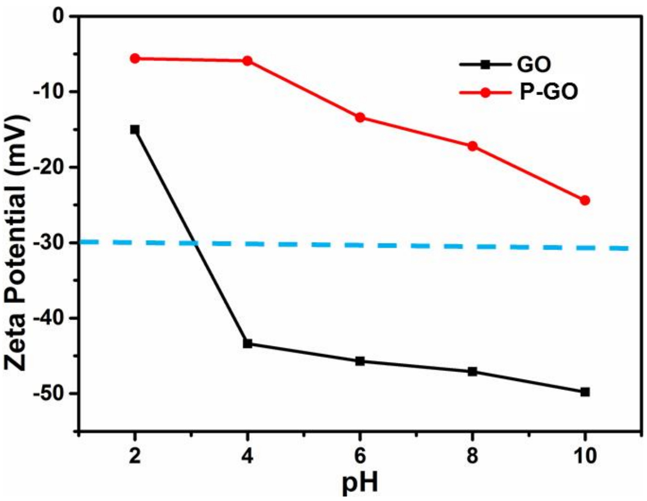

Supplementary Figure 17. The zeta potentials of GO and P-GO aqueous dispersion measured in a pH range of 2-10.

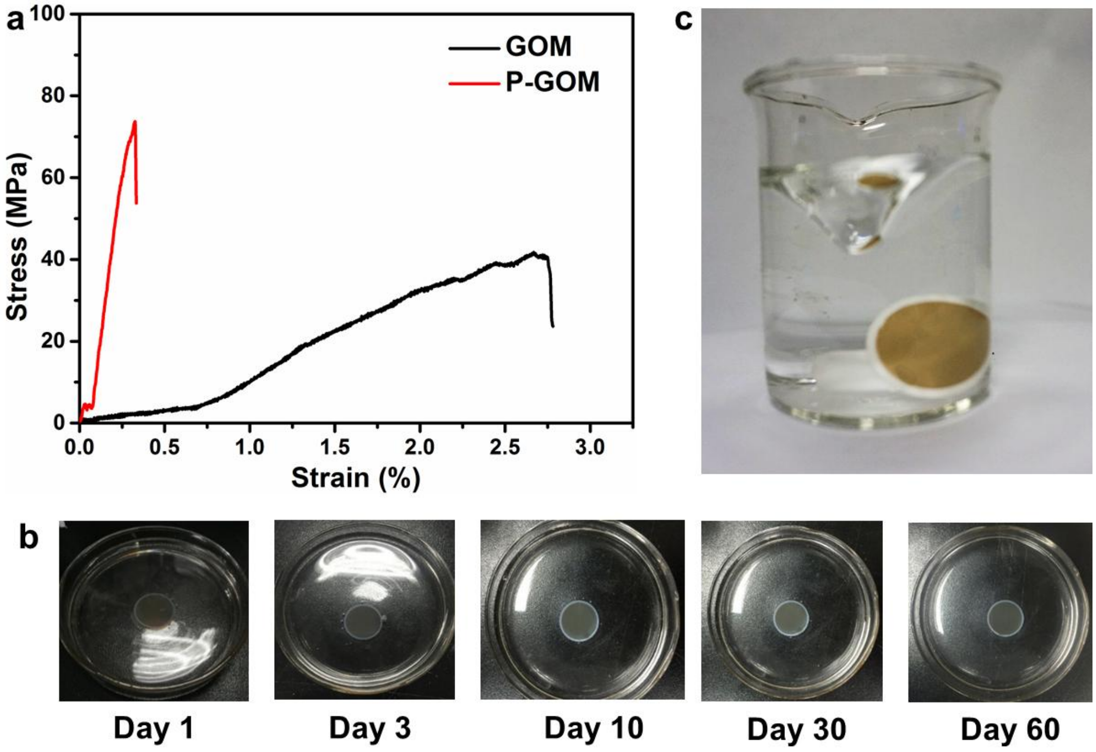

Supplementary Figure 18. Mechanical performance and water stability of P-GOM. a) The typical strain-stress curves of GOMs and P-GOMs. The P-GOM remained stable b) after immersing in water for 60 days, c) under a strong stirring.

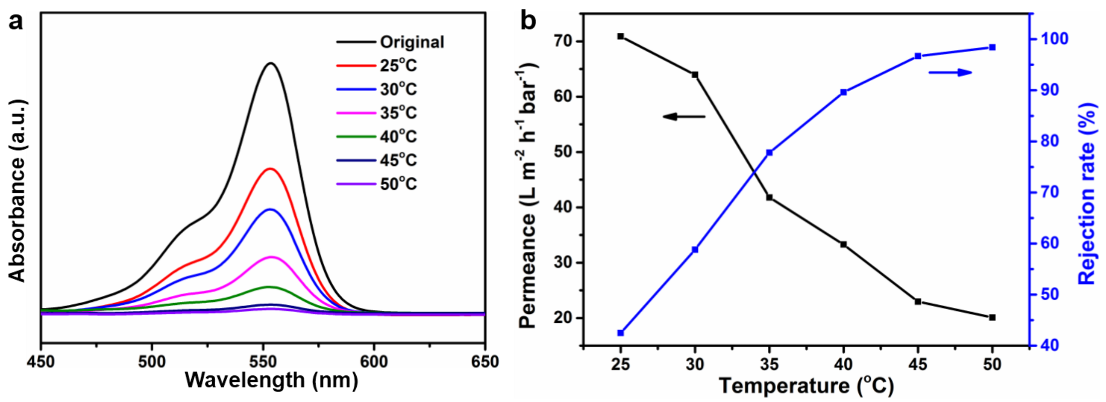

Supplementary Figure 19. The separation performance of 0.8 μm P-GOMs. a) The UV-vis absorption spectra before and after filtering RB from 25℃ to 50℃ using the 0.8 μm P-GOMs. b) Temperature dependent permeance and rejection rate of the 0.8 μm P-GOMs for RB. In this work, the thickness of P-GOMs is fixed at 1.1 μm in order to achieve a larger temperature-response gating ratio (Fig. 3d in manuscript) and realize the rejection rate of almost 100% for RB, thus improving the precision of mixed molecules gradient separation. However, separation membranes exhibit a trade-off between permeability and selectivity[5]. Therefore, we can improve the permeance by sacrificing a little selectivity of P-GOMs by using a thinner membrane. If we decreased the membrane thickness to 0.8 μm, the rejection rate slightly decreased from near 100% (1.1 μm) to 98.4% for RB as shown in Supplementary Fig. 19b. But its permeance significantly increased from 2.43 to 20.10 L m^{-2} h^{-1} bar^{-1}. According to the rejection rates for RB, the P-GOMs belong to nanofiltration-ranged separation membranes[6]. The permeance of 0.8 μm P-GOMs we reported is two to four times higher than commercial nanofiltration membranes of about 5-10 L m^{-2} h^{-1} bar^{-1}.[7-9]

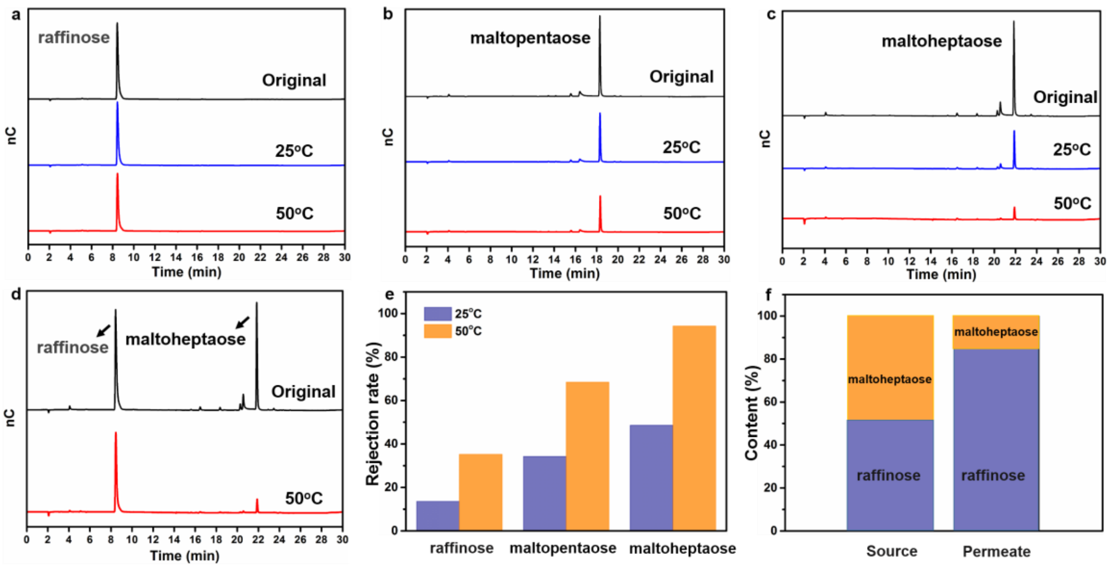

Supplementary Figure 20. P-GOMs for raffinose, maltopentaose and maltoheptaose separation. a-c) The ion chromatogram of the original feed solution and the permeate obtained at 25℃ and 50℃ using raffinose, maltopentaose and maltoheptaose as the single solute. d) The ion chromatogram of the original feed solution and the permeate obtained at 50℃ using a mixture of raffinose and maltoheptaose. e) The rejection rates of P-GOMs for raffinose, maltopentaose and maltoheptaose at 25℃ and 50℃. f) Source and permeate content of a mixture of raffinose and maltoheptaose. We further chose three kinds of saccharides, raffinose ($C_{18}H_{32}O_{16}$), maltopentaose ($C_{30}H_{52}O_{26}$) and maltoheptaose ($C_{42}H_{72}O_{36}$), to perform the separation experiments. Raffinose, maltopentaose and maltoheptaose are non-ionic solutes with hydrated diameter of 1.32 nm, 1.52 nm and 1.96 nm, respectively[10]. Firstly, the single-solute aqueous solutions of raffinose, maltopentaose and maltoheptaose were used as the feed solutions. As shown in Supplementary Fig. 20, the P-GOMs showed rejection rates of 13.6% for raffinose, 34.3% for maltopentaose and 48.6% for maltoheptaose at 25 ℃. When the temperature was 50 ℃, the rejection rates increased to 35.3% for raffinose, 68.4% for maltopentaose and 94.3% for maltoheptaose, respectively. At 50 ℃, since the P-GOMs demonstrated a relatively high rejection rate for maltoheptaose and a low rejection

rate for raffinose, we supposed that we could separate raffinose and maltoheptaose effectively. Subsequently, a mixture of raffinose and maltoheptaose (50:50% by weight) was filtered at 50 ℃. And as shown in Supplementary Figs. 20d and 20f, the mass content of raffinose in the permeate increased to 84%, indicating that the P-GOMs had a high selectivity towards raffinose and maltoheptaose. Therefore, the P-GOMs have the performance of reliability and high separation resolution.

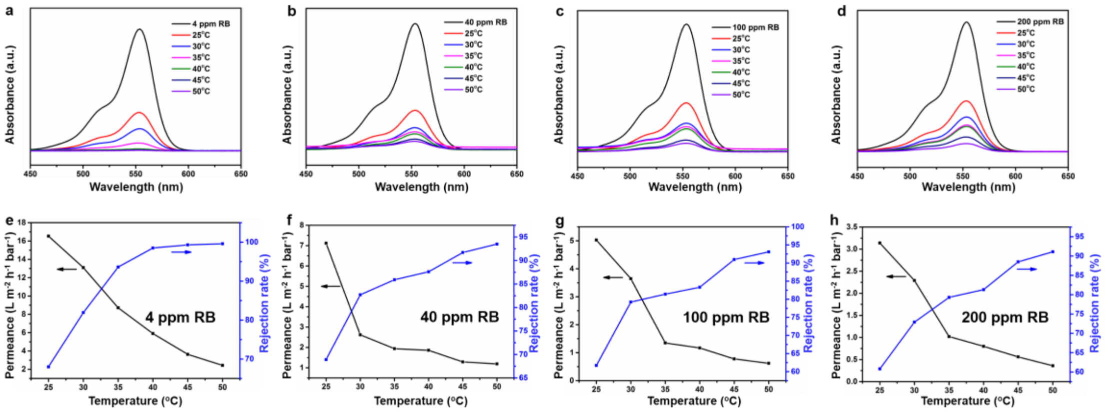

Supplementary Figure 21. The separation performance of P-GOMs for RB solution with different concentration. a-d) The UV-vis absorption spectra before and after filtering RB from 25℃ to 50℃ with the concentrations of 4 ppm, 40ppm, 100ppm and 200 ppm. e-h) Temperature dependent permeances and rejection rates of P-GOMs for 4 ppm, 40ppm, 100ppm and 200 ppm RB.

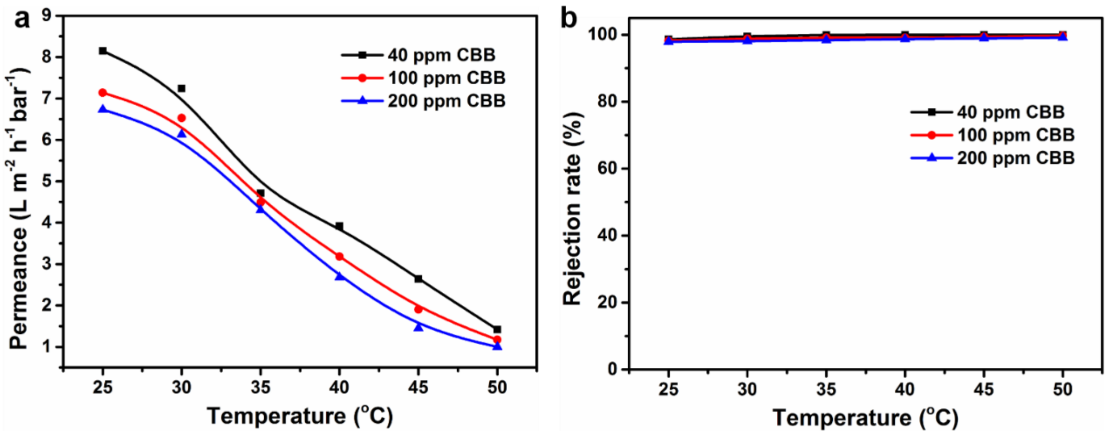

Supplementary Figure 22. The separation performance for CBB solution with different concentration. a) Temperature dependent permeances of P-GOMs for 40 ppm, 100ppm and 200 ppm CBB. b) Temperature dependent rejection rates of P-GOMs for 40 ppm, 100ppm and 200 ppm CBB. To explore how the P-GOMs would perform when using a more concentrated feed solution, we chose RB and CBB as examples to carry out the experiments. As shown in Supplementary Fig. 21, the permeance decreased with the RB concentration increasing from 4 mg/L (ppm) to 200 ppm. The rejection rates for these four concentrations of RB were 99.6%, 93.5%, 93.1% and 91.1%, respectively. It can be seen that there was only a slight rejection rate decrease for RB with the increased concentration. For CBB, the anionic dye, the rejection rates were ~100%, 99.7% and 99.1% using 40 ppm, 100 ppm and 200 ppm feed solutions, and the permeance variation was small (Supplementary Fig. 22). Therefore, the P-GOMs exhibited a good separation performance for a wide concentration range.

Supplementary Table 1. Reaction enthalpies (ΔH_{298}, kJ mol^{-1}) for the reactions shown in two reaction sets (1a-4a) and (1b-4b).

**Reaction**	$\Delta \boldsymbol{H}_{\mathbf{298}}$**(**set 1a-4a**)**	$\Delta \boldsymbol{H}_{\mathbf{298}}$**(**set 1b-4b**)**
(1)	50.9	–65.2
(2)	20.8	–154.6
(3)	–158.3	–254.5
(4)	–262.7	–396.0

Supplementary Table 2 The rejection rates and permeance of P-GOMs for molecules with different sizes at 25℃ and 50℃.

	Mw ($g\ mol^{-1}$)	size (nm)	concentration (mg/L)	analyte charge	rejection rate (%)		Permeance ($L\ m^{-2}\ h^{-1}\ bar^{-1}$)	
					25℃	50℃	25℃	50℃
Cu^{2+}	63.55	0.8	100.0	+	7.9	16.7	6.71	1.14
$[Fe(CN)_6]^{3-}$	212.00	0.9 × 0.9	40.0	−	12.5	48.2	10.24	2.00
RB	479.01	1.8 × 1.4	4.0	+	68.2	100.0	16.53	2.43
CBB	826.97	2.7 × 1.8	40.0	−	98.0	100.0	8.15	1.42
Cyt. c	12,800	2.5 × 2.5 × 3.7	100.0	−	99.8	100.0	7.98	1.31

Supplementary Note 1

The GO weight fraction (ω) in P-GO nanocomposites could be decided by Supplementary Equation (1)[11]:

$$\omega = \frac{M - M_{PNIPAM}}{M_{GO} - M_{PNIPAM}} \quad (1)$$

Where M , M_{PNIPAM} and M_{GO} represent the weight loss of P-GO, PNIPAM and GO between 100℃ and 500℃. As can be calculated the TGA and DTG data from Supplementary Fig. 13, the values of M , M_{PNIPAM} and M_{GO} are 77.52%, 94.60% and 37.42%, thus the GO weight fraction of P-GO is calculated to be approximate 30%. Thus, the mass ratio of GO and PNIPAM to fabricate the physically blended GO/PNIPAM membrane as the control experiment was 3:7. In fact, the positively temperature-responsive property of the GO blending with PNIPAM membranes was independent of the mass ratio of GO and PNIPAM as shown in Supplementary Fig. 14.

Up to now, several papers have reported the negatively temperature-responsive property of membranes modified by PNIPAM[12-14]. However, the negatively gating behaviors shown by them were all based on diffusional permeation experiments instead of hydraulic permeability. Since PNIPAM would become hydrophobic when the temperature is above its LCST, the negatively gating behaviors in diffusional permeation experiments may be caused by the wettability variation which changes from hydrophilic to hydrophobic when the temperature arises above LCST. In this paper, we performed hydraulic permeability experiments with a pressure of 100 kPa. So the change of membrane channel size dominated the gating property rather than

the wettability variation. To the best of our knowledge, no negatively temperature-responsive gating membranes modified with PNIPAM applied to hydraulic permeability applications has been reported to date.

Supplementary Note 2

The polymerization reaction mechanisms for the two possibilities involve the following reactions:

$[C_{36}H_{16}\text{-}CH_3]\bullet + NIPAM \rightarrow C_{36}H_{16}\text{-}CH_3\text{-}NIPAM\bullet$ (1a)

$[C_{36}H_{16}\text{-}CH_3]\bullet + 2NIPAM \rightarrow C_{36}H_{16}\text{-}CH_3\text{- }[NIPAM]_2\bullet$ (2a)

$[C_{36}H_{16}\text{-}CH_3]\bullet + 3NIPAM \rightarrow C_{36}H_{16}\text{-}CH_3\text{- }[NIPAM]_3\bullet$ (3a)

$[C_{36}H_{16}\text{-}CH_3]\bullet + 4NIPAM \rightarrow C_{36}H_{16}\text{-}CH_3\text{- }[NIPAM]_4\bullet$ (4a)

$2[C_{36}H_{16}\text{-}CH_3]\bullet + NIPAM \rightarrow C_{36}H_{16}\text{-}CH_3\text{-}NIPAM\text{-}CH_3\text{-}C_{36}H_{16}$ (1b)

$2[C_{36}H_{16}\text{-}CH_3]\bullet + 2NIPAM \rightarrow C_{36}H_{16}\text{-}CH_3\text{-}(NIPAM)_2\text{-}CH_3\text{-}C_{36}H_{16}$ (2b)

$2[C_{36}H_{16}\text{-}CH_3]\bullet + 3NIPAM \rightarrow C_{36}H_{16}\text{-}CH_3\text{-}(NIPAM)_3\text{-}CH_3\text{-}C_{36}H_{16}$ (3b)

$2[C_{36}H_{16}\text{-}CH_3]\bullet + 4NIPAM \rightarrow C_{36}H_{16}\text{-}CH_3\text{-}(NIPAM)_4\text{-}CH_3\text{-}C_{36}H_{16}$ (4b)

Supplementary Note 3

We suppose that, for the negatively temperature-responsive P-GOMs, when the temperature increased above its LCST the first case and second case discussed in main text played primary roles to change the distance between GO sheets as shown in Figure. 4a. But we admit that the responsive behavior of PNIPAM chain as Supplementary Fig. 11 shows also exists, however, has much less influence than the

first case and second case discussed in the main text.

Supplementary Note 4

In practical applications, the mechanical and structural stability of membranes is of crucial importance, so tensile experiments were performed (Supplementary Fig. 18a). The tensile strength of P-GOMs reached up to 75 MPa, which was almost 2 times higher than that of GOMs because of the effective covalent linkage between GO sheets by the polymer chains. The value was higher than those free-standing graphene-based membranes used for separation in previous studies[15]. Moreover, the P-GOMs were highly stable in water as shown in Supplementary Fig. 18b. After immersing P-GOMs in water for 60 days, they remained intact even under a strong stirring (Supplementary Fig. 18c). According to the knowledge of colloidal chemistry, the particle could disperse if the absolute values of zeta potentials were larger than 30 mV, thus the P-GOMs could remain stable in water for a long time because of the low absolute value of zeta potentials (Supplementary Fig. 17)[16,17]. Therefore, the good mechanical performance and water stability endow the P-GOMs with promising practicality for durable temperature-response gating and separation applications.

Supplementary References

1 Gu, W. & Tripp, C. P. Reaction of silanes in supercritical CO_2 with TiO_2 and Al_2O_3. *Langmuir* **22**, 5748-5752 (2006).

2 Kundu, A., Nandi, S., Das, P. & Nandi, A. K. Fluorescent graphene oxide via polymer grafting: an efficient nanocarrier for both hydrophilic and hydrophobic drugs. *ACS Appl. Mater. Inter.* **7**, 3512-3523 (2015).

3 Pei, S. & Cheng, H.-M. The reduction of graphene oxide. *Carbon* **50**, 3210-3228 (2012).

4 Wang, Y. *et al.* Graphene‐Directed Supramolecular Assembly of Multifunctional Polymer Hydrogel Membranes. *Adv. Funct. Mater.* **25**, 126-133 (2015).

5 Park, H. B., Kamcev, J., Robeson, L. M., Elimelech, M. & Freeman, B. D. Maximizing the right stuff: The trade-off between membrane permeability and selectivity. *Science* **356**, eaab0530 (2017).

6 Wang, X.-L., Shang, W.-J., Wang, D.-X., Wu, L. & Tu, C.-H. Characterization and applications of nanofiltration membranes: State of the art. *Desalination* **236**, 316-326 (2009).

7 Akbari, A. *et al.* Large-area graphene-based nanofiltration membranes by shear alignment of discotic nematic liquid crystals of graphene oxide. *Nat. Commun.* **7**, 10891 (2016).

8 Boussu, K. *et al.* Characterization of polymeric nanofiltration membranes for systematic analysis of membrane performance. *J. Membr. Sci.* **278**, 418-427 (2006).

9 Agenson, K. O., Oh, J.-I. & Urase, T. Retention of a wide variety of organic pollutants by different nanofiltration/reverse osmosis membranes: controlling parameters of process. *J. Membr. Sci.* **225**, 91-103 (2003).

10 Schultz, S. G. & Solomon, A. Determination of the effective hydrodynamic radii of small

molecules by viscometry. *J. Gen. Physiol.* **44**, 1189-1199 (1961).

11 Wan, S. *et al.* Use of synergistic interactions to fabricate strong, tough, and conductive artificial nacre based on graphene oxide and chitosan. *ACS nano* **9**, 9830-9836 (2015).

12 Chu, L. Y., Niitsuma, T., Yamaguchi, T. & Nakao, S. i. Thermoresponsive transport through porous membranes with grafted PNIPAM gates. *AlChE J.* **49**, 896-909 (2003).

13 Li, Y. *et al.* Thermoresponsive gating characteristics of poly (N-isopropylacrylamide)-grafted porous poly (vinylidene fluoride) membranes. *Ind. Eng. Chem. Res.* **43**, 2643-2649 (2004).

14 Schepelina, O. & Zharov, I. PNIPAAM-modified nanoporous colloidal films with positive and negative temperature gating. *Langmuir* **23**, 12704-12709 (2007).

15 Liu, N. *et al.* Ultralight free-standing reduced graphene oxide membranes for oil-in-water emulsion separation. *J. Mater. Chem. A* **3**, 20113-20117 (2015).

16 Yang, J. & Gunasekaran, S. Electrochemically reduced graphene oxide sheets for use in high performance supercapacitors. *Carbon* **51**, 36-44 (2013).

17 Everett, D. Basic Principles of Colloid ScienceRoyal Society of Chemistry. *Cambridge, UK* (1988).
